# Apolipoproteins and high-density lipoprotein phospholipids as indicators of atherosclerotic cardiovascular disease in Nigeria

**DOI:** 10.4102/ajlm.v14i1.2942

**Published:** 2025-12-17

**Authors:** Promise C. Nwaejigh, Maria O. Ebesunun, Stephen S. Udofia, Adebusola A. Shakunle

**Affiliations:** 1 Department of Medical Laboratory Science, School of Allied Health Sciences, Babcock University, Ilishan-Remo, Nigeria; 2 Department of Medical Laboratory Science, Faculty of Basic Medical Sciences, Olabisi Onabanjo University, Sagamu, Nigeria; 3 Department of Obstetrics and Gynaecology, Lagos State University College of Medicine, Ikeja, Nigeria

**Keywords:** atherosclerotic cardiovascular disease, apolipoprotein A1, apolipoprotein B, apolipoprotein B/A1 ratio, HDL phospholipids, lipid profile

## Abstract

**Background:**

Altered apolipoproteins and high-density lipoprotein (HDL) phospholipids are linked to premature atherosclerotic cardiovascular disease (ASCVD).

**Objective:**

This study investigated associations between plasma apolipoprotein A1, apolipoprotein B, HDL phospholipids, and ASCVD risk in Nigeria, assessing their potential as early diagnostic markers.

**Methods:**

This cross-sectional case-control study was conducted from November 2021 to November 2022 at Lagos State University Teaching Hospital in Nigeria. Atherosclerotic cardiovascular disease patients and healthy controls were randomly selected. The plasma apolipoprotein A1 and B levels were determined via a sandwich enzyme-linked immunosorbent assay, and the lipid profile was measured via spectrophotometry. Statistical analyses included *t*-tests, analysis of variance, analysis of covariance, and Pearson’s correlation.

**Results:**

In total, 172 confirmed ASCVD patients (mean age: 54.01 ± 8.70 years) and 55 healthy controls (mean age: 44.55 ± 11.60 years) were included in the analyses. Compared with the control values, ASCVD patients showed significantly elevated apolipoprotein B, apolipoprotein B/A1 ratio, atherogenic lipid indices, total cholesterol, low-density lipoprotein cholesterol, non-HDL cholesterol, and triglycerides (*p* ≤ 0.001). In contrast, plasma HDL phospholipids, apolipoprotein A1, and HDL cholesterol were markedly lower (*p* ≤ 0.001).

**Conclusion:**

These findings indicate that altered apolipoproteins and HDL phospholipids are associated with premature ASCVD risk, with the apolipoprotein B/A1 ratio emerging as a superior marker for disease stratification.

**What this study adds:**

This study identifies the apolipoprotein B/A1 ratio as a strong early marker of ASCVD risk in Nigeria.

## Introduction

Atherosclerotic cardiovascular disease (ASCVD) represents a significant health challenge globally, with a rising incidence in Nigeria and other developing regions.^1,2^ High-density lipoprotein (HDL) and its associated apolipoproteins, namely apolipoprotein A1 (Apo A1) and apolipoprotein B (Apo B), along with HDL phospholipids, have garnered considerable attention for their intricate involvement in ASCVD progression.

Although research has explored lipid dysregulation and atherogenesis extensively, most of these studies have focused predominantly on populations from Western countries,^3,4^ and Asian populations.^5,6^ However, it is imperative to recognise the unique genetic, environmental, cultural dietary habits and lifestyle factors that contribute to ASCVD susceptibility in diverse ethnic groups, particularly African populations.

Nigeria, as a key part of Africa, is experiencing a growing ASCVD epidemic as a result of urbanisation, Westernised diets and changing lifestyles.^7,8^ Despite the increase in ASCVD in Nigeria and Africa, comprehensive studies are scarce on the specific lipid profiles associated with atherosclerosis.

Apolipoprotein A1, the major protein component of HDL, is integral to the process of reverse cholesterol transport,^9,10^ exerting anti-inflammatory and antioxidant effects that confer protection against ASCVD.^11,12^ In contrast, elevated levels of Apo B, which is found predominantly on low-density lipoprotein particles, are associated strongly with increased ASCVD risk,^13,14^ reflecting the proatherogenic properties of low-density lipoprotein cholesterol (LDLC).^15^

The Apo B/A1 ratio is used increasingly to assess cardiovascular risk. A lower ratio indicates a better balance between atherogenic and protective lipoproteins, which correlates with a lower risk of ASCVD.^16^ Recent studies have also emphasised this ratio as a more accurate predictor of cardiovascular events than traditional lipid measurements.^17^ Advances in genomics have revealed that genetic variations influence Apo A1 and Apo B levels, affecting ASCVD.^18,19,20^

Furthermore, the composition and functionality of HDL, particularly its phospholipid content, have garnered attention for their potential impact on ASCVD risk. HDL phospholipids, comprising a diverse array of molecular species, contribute significantly to HDL functionality, modulating its anti-inflammatory, antioxidative, and vasoprotective properties.^21,22^ Alterations in the composition and functionality of HDL phospholipids have been implicated in ASCVD.^23,24^

Higher levels of specific HDL phospholipids are linked to a lower risk of major adverse cardiovascular events, suggesting that they could enhance traditional lipid markers for better risk stratification.^23,25^ Emerging evidence suggests that HDL phospholipid composition may be a cardiovascular risk biomarker.^26^

This study assessed atherogenic lipid profiles in Nigerian patients with ASCVD, with a specific focus on apolipoproteins and HDL phospholipids. This study examined plasma Apo A1, Apo B, total and HDL phospholipids, and lipid profiles in ASCVD patients.

## Methods

### Ethical considerations

This study was approved by the Health Research and Ethics Committee of Lagos State University Teaching Hospital in Nigeria (reference number: LREC/06/10/1706). Before participation, all individuals provided written informed consent. All procedures followed the ethical principles of the 2024 Declaration of Helsinki. To ensure anonymity, a barcode system was employed, and all data were handled with strict confidentiality. The collected data were used solely for this study.

### Study design

This cross-sectional case-control study determined a sample size of 138 based on prevalence data from a previous regional study,^27^ which reported an 8% – 12% prevalence of cardiovascular disease in sub-Saharan African countries.^28^ In total, 172 confirmed ASCVD patients, aged 20 years to 64 years, were enrolled. Participants were selected using a simple random sampling technique at the Lagos State University Teaching Hospital, Lagos, Nigeria. We enrolled participants diagnosed with hypertensive heart disease with significant atherosclerosis, ischaemic heart disease (IHD), myocardial infarction (MI), ischaemic stroke, or congestive heart failure linked to prior IHD or MI. Recruitment took place during routine outpatient visits at the Cardiology Clinic, Lagos State University Teaching Hospital, Lagos, Nigeria, from November 2021 to November 2022. Eligible participants were identified through consecutive clinic record reviews and were enrolled at most six months post-diagnosis, with clinical stability confirmed by no acute cardiovascular events (e.g. MI or stroke) in the prior three months, as verified by a cardiologist and medical records. The IHD group comprised patients with stable conditions, such as stable angina, without a documented MI. The MI group included those with a prior MI, defined per the 2012 Universal Definition of Myocardial Infarction,^29^ based on clinical signs of ischaemia, elevated cardiac biomarkers (e.g. troponin) and echocardiographic findings. To minimise confounding effects on lipid-related biomarkers, we excluded patients on lipid-lowering therapy at recruitment or within the prior six months, as well as those on hormonal therapy, pregnant women, or individuals who declined participation. In our setting, some patients were not on lipid-lowering therapy owing to clinical contraindications (e.g. statin intolerance) or non-adherence, verified through medical records and patient interviews. We also excluded patients with acute MI, stroke, or other cardiovascular events within three months before sampling, to focus on early-stage ASCVD and to support our aim of evaluating plasma biomarkers for early detection.

The control group consisted of 55 healthy volunteers with no history of chronic or degenerative diseases, recruited from relatives of patients and staff at the Lagos State University Teaching Hospital. All participants completed a standardised, structured paper questionnaire (Online Supplementary Questionnaire) to capture general characteristics, inclusion/exclusion criteria, and potential confounding factors that could influence study outcomes. Control participants were further evaluated for clinical examination, and basic laboratory screening, including fasting plasma glucose, lipid profile, and blood pressure measurements, to confirm their apparently healthy status and exclude subclinical chronic or metabolic conditions

### Blood sample collection

Blood samples from ASCVD patients were collected within 0–6 months of diagnosis to capture early-stage disease, consistent with the aim of the study to evaluate apolipoprotein A1, apolipoprotein B, and HDL phospholipids as potential early diagnostic markers. Approximately 7 mL of venous blood was drawn from each participant after an overnight fast (8 h – 12 h). Samples were collected in potassium-ethylenediaminetetraacetic acid (1 mg/mL) tubes, maintained at 4 °C, and processed within two hours. Plasma was separated by centrifugation at 3000 rpm for 5 min, aliquoted into labelled vials, and stored at –20 °C until analysis, conducted within 1–12 months. The use of potassium-ethylenediaminetetraacetic acid minimised lipid peroxidation and was appropriate for apolipoprotein and phospholipid assays. Although minor effects on total cholesterol and LDLC might occur, consistent use across all samples ensured data comparability and analyte stability.

### Biochemical analysis

Lipid profile parameters (total cholesterol, high-density lipoprotein cholesterol [HDLC] and triglycerides), were measured via commercial kits (Randox Laboratories Ltd., Crumlin, Northern Ireland, United Kingdom).

Total cholesterol and HDLC (following the removal of all non-HDL lipid fractions with phosphotungstic acid and magnesium ions) were estimated using the methods of Allain et al.^30^ Triglycerides were measured via the method developed by Bucolo and David.^31^ The calculation of LDLC was performed using the formula provided by Friedewald, Levy and Fredrickson,^32^ selected for its validation, cost-effectiveness, and suitability in resource-limited settings such as sub-Saharan Africa, where direct methods (e.g. ultracentrifugation or the Martin-Hopkins equation) are often impractical. All samples had triglyceride levels below 4.52 mmol/L, confirming the validity of the equation. Based on these measurements, we further evaluated derived lipid indices, including LDLC, non-HDLC, Castelli’s risk indices (CRI-I, CRI-II), atherogenic coefficient (AC), and atherogenic index of plasma (AIP), using standard lipid panel measurements (total cholesterol [TC], triglycerides, HDLC). Formulas and clinical relevance are provided in Online Supplementary Table 1.^32,33,34^

**TABLE 1:** General characteristics and biochemical parameters of study population at Lagos State University Teaching Hospital, Lagos, Nigeria, between November 2021 and November 2022.

Variable	ASCVD patients (*n* = 172)		Controls (*n* = 55)		*t*	*p*	Cohen’s *d*	*r* ^2^
	Mean	s.d.	Mean	s.d.				
Age (years)	54.01	8.70	44.55	11.60	6.446	< 0.001*	0.999	0.156
Total cholesterol (mmol/L)	4.90	0.72	3.72	0.41	11.516	< 0.001*	1.784	0.371
Triglycerides (mmol/L)	1.33	0.35	0.92	0.26	7.860	< 0.001*	1.218	0.215
HDL cholesterol (mmol/L)	1.06	0.10	1.23	0.09	−10.570	< 0.001*	−1.637	0.332
LDL cholesterol (mmol/L)	3.23	0.62	2.07	0.37	13.104	< 0.001*	2.030	0.433
Non-HDL cholesterol (mmol/L)	3.84	0.73	2.49	0.39	13.072	< 0.001*	2.025	0.432
Castelli’s Risk Index I	4.66	0.85	3.03	0.33	13.753	< 0.001*	2.130	0.457
Castelli’s Risk Index II	3.09	0.73	1.70	0.32	13.772	< 0.001*	2.133	0.457
Atherogenic coefficient	3.64	0.85	2.03	0.33	13.716	< 0.001*	2.125	0.455
Atherogenic index of plasma	0.44	0.13	0.22	0.12	11.346	< 0.001*	1.758	0.364
Apolipoprotein A1 (ng/mL)	40.09	7.09	53.71	11.26	−10.609	< 0.001*	−1.643	0.333
Apolipoprotein B (ng/mL)	1038.23	153.91	959.87	197.09	3.060	0.002*	0.474	0.040
Apolipoprotein B/A1	26.34	4.75	18.09	3.06	12.085	< 0.001*	1.872	0.394
Total phospholipids (mg/dL)	162.48	19.61	164.35	11.78	−0.687	0.493	−0.106	0.002
HDL phospholipids (mg/dL)	81.21	11.78	112.45	13.89	−16.377	< 0.001*	−2.537	0.544

Note: Cohen’s *d* represents standardised mean difference; *r*^2^ indicates proportion of variance explained by group; *t*-value from Student’s *t*-test.

ASCVD, atherosclerotic cardiovascular disease; HDL, high-density lipoprotein; LDL, low-density lipoprotein; s.d., standard deviation.

* , indicates significant difference between ASCVD patients and controls (*p* < 0.05).

Plasma total phospholipids were analysed via the Wako Phospholipid C Assay Kit (Fujifilm Wako Pure Chemical Industries, Ltd., Osaka, Japan), which employs the N-ethyl-N-(2-hydroxy-3-sulfopropyl) 3,5-dimethoxy aniline sodium salt method.^35^ High-density lipoprotein phospholipids were assayed via this method after treatment of participants’ plasma with phosphotungstic acid and magnesium ions (to remove all non-HDL phospholipids fractions).^36^

The plasma levels of human Apo A1 and Apo B in the participants were determined using the sandwich enzyme-linked immunosorbent assay technique,^37^ with assay kits sourced from Elabscience Biotechnology Limited, Wuhan, China. Analyses were performed on a Bio-Rad iMark™ Microplate Reader (Bio-Rad Laboratories, Hercules, California, United States) at 450 nm. The system performs optical calibration and data processing automatically, following the manufacturer’s instructions. Quality control sera were included in each batch to ensure analytical precision and reliability.

Sandwich enzyme-linked immunosorbent assay was selected for apolipoprotein analysis because of its high specificity, sensitivity and practical advantages over Western blotting and radioactive immunoassays. Spectrophotometry was chosen for lipid profiling because of its efficiency and cost-effectiveness, providing direct measurement of lipid fractions. Chromatographic methods, although precise, require more resources and technical skill, making spectrophotometry a more accessible option for routine analysis in sub-Saharan Africa.

### Data analysis

Statistical analyses were conducted using IBM SPSS Statistics (version 21, IBM Corp., Armonk, New York, United States). Data completeness and normality were verified, with continuous variables reported as means ± standard deviation, and categorical variables as frequencies (percentages). Normality of lipid and apolipoprotein outcomes (Apo A1, Apo B, HDL phospholipids, TC, LDLC, HDLC, triglycerides, non-HDLC, CRI-I, CRI-II, AC, AIP) was assessed using Shapiro–Wilk tests and Q–Q plots (*n* = 227). Non-normal variables were log-transformed. Levene’s test confirmed homogeneity of variances (*p* > 0.05).

Group differences (ASCVD vs control) were analysed using independent samples *t*-tests and one-way analysis of variance with Bonferroni correction (*p* < 0.001). Effect sizes were reported as Cohen’s *d, r*^2^, and partial *η*^2^. Separate analysis of covariance models, with age as the covariate, were used for each lipid and apolipoprotein outcome owing to its significant correlation with outcomes (*p* < 0.05, Pearson’s correlation; Online Supplementary Table 2)

Other potential confounders (sex, BMI, smoking, hypertension) were non-significant (*p* > 0.05) and excluded, along with diabetes and lipid-lowering medication use.

Analysis of covariance assumptions were met: linearity (via scatterplots and Pearson’s correlation), homogeneity of regression slopes (group × covariate interaction, *p* > 0.05), normality of residuals (Shapiro–Wilk and Q–Q plots), homoscedasticity (Breusch-Pagan test, *p* > 0.05), and independence of residuals (Durbin–Watson ≈ 2.0). Age-adjusted means and partial eta-squared (*η*^2^) values for lipid parameters are presented in Online Supplementary Table 3 to Online Supplementary Table 9.

## Results

### General characteristics of the study population

A total of 172 confirmed ASCVD patients (73 men, 99 women) aged 20–64 years were enrolled, including those with IHD (*n* = 40), ischaemic stroke (*n* = 25), hypertensive heart disease with significant atherosclerosis (*n* = 45), MI (*n* = 20), and congestive heart failure with a history of IHD or MI (*n* = 42). The control group comprised 55 healthy volunteers (24 men, 31 women) aged 20–64 years. Atherosclerotic cardiovascular disease patients showed significantly higher mean plasma TC, LDLC, non-HDLC, and triglycerides levels than controls (all *p* < 0.001, Table 1). Similarly, all conventional lipid indices (CRI-I, CRI-II, AC, and AIP) (*p* < 0.001), Apo B (*p* = 0.002), and the Apo B/A1 ratio (*p* < 0.001) were elevated significantly.

Conversely, mean plasma HDLC, Apo A1, and HDL phospholipid levels were significantly lower in ASCVD patients (all *p* < 0.001). After adjusting for age using analysis of covariance, significant differences remained in all lipid and apolipoprotein parameters between ASCVD patients and controls. Full statistical details are provided in Online Supplementary Table 3 to Online Supplementary Table 10.

### Conventional lipid profiles of atherosclerotic cardiovascular disease subclasses

Significant increases were observed in the mean plasma levels of TC, LDLC, non-HDLC, and triglycerides (*p* < 0.001) across ASCVD subclasses and controls (Table 2). Similarly, all conventional lipid indices (CRI-I, CRI-II, AC, and AIP) showed marked elevations (*p* < 0.001). In contrast, plasma HDLC levels were significantly reduced (*p* < 0.001) across the ASCVD subclasses and controls. Patients with IHD and MI had the highest levels of atherogenic lipids (triglycerides and TC) and lipoproteins (LDLC and non-HDLC), with mean values significantly elevated compared to controls and other ASCVD subgroups (*p* < 0.001). These groups also presented the lowest HDLC levels (*p* < 0.001), indicating a more atherogenic lipid profile.

**TABLE 2:** Conventional lipid profile and indices in atherosclerotic cardiovascular disease subclasses and controls at Lagos State University Teaching Hospital, Lagos, Nigeria, between November 2021 and November 2022.

Variables	HHD (*n* = 45)		Stroke (*n* = 25)		IHD (*n* = 40)		MI (*n* = 20)		HF (*n* = 42)		Controls (*n* = 55)		*F*	*p*	Partial *η*^2^
	Mean	s.d.	Mean	s.d.	Mean	s.d.	Mean	s.d.	Mean	s.d.	Mean	s.d.			
Total cholesterol (mmol/L)	4.63*	0.70	4.63*	0.47	5.31*	0.74	5.45*	0.56	4.69*	0.62	3.72	0.41	45.529	< 0.001*	0.495
Triglyceride (mmol/L)	1.16*	0.23	1.16*	0.22	1.61*	0.31	1.60*	0.23	1.19*	0.30	0.92	0.26	36.570	< 0.001*	0.477
HDL cholesterol (mmol/L)	1.09*	0.12	1.06*	0.08	1.03*	0.07	0.99*	0.09	1.10*	0.10	1.23	0.09	29.972	< 0.001*	0.416
LDL cholesterol (mmol/L)	3.02*	0.62	2.99*	0.42	3.54*	0.63	3.73*	0.52	3.07*	0.52	2.07	0.37	51.174	< 0.001*	0.536
Non-HDL cholesterol (mmol/L)	3.54*	0.70	3.57*	0.47	4.28*	0.73	4.46*	0.58	3.59*	0.58	2.49	0.39	58.156	< 0.001*	0.567
Castelli’s Risk Index I	4.30*	0.83	4.39*	0.54	5.17*	0.76	5.57*	0.82	4.28*	0.54	3.03	0.33	74.054	< 0.001*	0.626
Castelli’s Risk Index II	2.84*	0.75	2.85*	0.50	3.45*	0.66	3.83*	0.72	2.80*	0.46	1.70	0.32	64.225	< 0.001*	0.592
Atherogenic coefficient	3.30*	0.83	3.39*	0.54	4.12*	0.77	4.57*	0.82	3.28*	0.54	2.03	0.33	71.690	< 0.001*	0.619
Atherogenic index of plasma	0.38*	0.12	0.39*	0.09	0.55*	0.09	0.57*	0.07	0.38*	0.11	0.22	0.12	57.939	< 0.001*	0.566

ASCVD, atherosclerotic cardiovascular disease; *F*-value, analysis of variance across ASCVD subclasses and controls; HDL, high-density lipoprotein; HF, heart failure; HHD, hypertensive heart disease; IHD, ischaemic heart disease; MI, myocardial infarction; *n*, number of participants; *p*-value, level of significance from analysis of variance; s.d., standard deviation; Partial *η*^2^, effect size from one-way analysis of variance.

* , *p* < 0.01 versus controls (post-hoc Welch’s *t*-test with Bonferroni correction for multiple comparisons).

### Apolipoproteins and high-density lipoprotein phospholipid levels in atherosclerotic cardiovascular disease subclasses

Significant increases in mean plasma Apo B, and the Apo B/A1 ratio (*p* < 0.001) were observed across ASCVD subclasses and controls (Table 3). Conversely, mean plasma Apo A1 and HDL phospholipid levels were significantly reduced (*p* < 0.001). The highest Apo B and Apo B/A1 ratios were found in patients with MI and IHD, who also had the lowest levels of Apo A1 and HDL phospholipids. The Apo B/A1 ratio proved to be a superior marker for ASCVD stratification compared to Apo A1 or Apo B alone.^38^ For instance, Apo B levels did not differ significantly when patients suffering from stroke and heart failure were compared with the control group (Online Supplementary Table 10), whereas the Apo B/A1 ratio showed statistically significant differences across all ASCVD subclasses when each subclass is compared with the control group (Online Supplementary Table 11).

**TABLE 3:** Apolipoproteins and high-density lipoprotein phospholipids in atherosclerotic cardiovascular disease subclasses and controls at Lagos State University Teaching Hospital, Lagos, Nigeria, between November 2021 and November 2022.

Variables	HHD (*n* = 45)		Stroke (*n* = 25)		IHD (*n* = 40)		MI (*n* = 20)		HF (*n* = 42)		Controls (*n* = 55)		*F*	*p*	Partial *η*^2^
	Mean	s.d.	Mean	s.d.	Mean	s.d.	Mean	s.d.	Mean	s.d.	Mean	s.d.			
Apolipoprotein A1 (ng/mL)	43.02*	7.47	41.49*	5.09	36.79*	5.05	34.50*	3.79	41.93*	7.50	53.71	11.26	29.976	< 0.001*	0.410
Apolipoprotein B (ng/mL)	1057.00*	77.02	961.00	83.67	1089.00*	31.48	1099.00*	108.96	986.00	166.78	959.00	197.08	5.596	< 0.001*	0.112
Apolipoprotein B/A1	24.83*	3.51	23.29*	2.29	29.75*	3.35	32.23*	4.72	23.72*	3.60	18.09	3.06	79.181	< 0.001*	0.643
Total Phospholipids (mg/dL)	167.16	22.71	164.40	6.79	160.48	18.85	161.15	8.57	158.88	21.68	164.35	11.78	1.193	0.314	0.027
HDL phospholipids (mg/dL)	88.00*	12.99	85.80*	7.28	73.20*	5.52	72.15*	5.51	83.14*	2.38	112.45	13.89	77.537	< 0.001*	0.637

ASCVD, atherosclerotic cardiovascular disease; *F*-value, analysis of variance across ASCVD subclasses and controls; HDL, high-density lipoprotein; HF, heart failure; HHD, hypertensive heart disease; IHD, ischaemic heart disease; MI, myocardial infarction; *n*, number of participants; *p*-value, level of significance from analysis of variance; s.d., standard deviation; Partial *η*^2^, effect size from one-way analysis of variance.

* , *p* < 0.01 versus controls (post-hoc Welch’s *t*-test with Bonferroni correction for multiple comparisons).

### Associations between apolipoproteins, high-density lipoprotein phospholipids and conventional lipid profiles

A significant negative correlation was observed between mean plasma Apo A1 levels and atherogenic lipids, including triglycerides (*r* = –0.386, *p* < 0.001), TC (*r* = –0.471, *p* < 0.001), LDLC (*r* = –0.483, *p* < 0.001), and non-HDLC (*r* = –0.500, *p* < 0.001) (Table 4). In contrast, Apo A1 showed a strong positive correlation with antiatherogenic HDLC (*r* = 0.386, *p* < 0.001).

**TABLE 4:** Association between apolipoproteins, high-density lipoprotein phospholipids and conventional lipid profiles at Lagos State University Teaching Hospital, Lagos, Nigeria, between November 2021 and November 2022.

Variables	*r*	*p*
Apo A1 (ng/mL) – Total cholesterol (mmol/L)	−0.471	< 0.001**
Apo A1 (ng/mL) – Triglyceride (mmol/L)	−0.451	< 0.001**
Apo A1 (ng/mL) – HDL cholesterol (mmol/L)	0.386	< 0.001**
Apo A1 (ng/mL) – LDL cholesterol (mmol/L)	−0.483	< 0.001**
ApoA1 (ng/mL) – Non-HDL cholesterol (mmol/L)	−0.500	< 0.001**
Apo B (ng/mL) – Total cholesterol (mmol/L)	0.179	0.007**
Apo B (ng/mL) – Triglyceride (mmol/L)	0.158	0.017*
Apo B (ng/mL) – LDL cholesterol (mmol/L)	0.164	0.013*
Apo B (ng/mL) – Non-HDL cholesterol (mmol/L)	0.182	0.006**
Apo B/A1 – Castelli’s Risk Index 1	0.626	< 0.001**
Apo B/A1 –- Castelli’s Risk Index 1I	0.601	< 0.001**
Apo B/A1 – Atherogenic coefficient	0.613	< 0.001**
Apo B/A1 – Atherogenic index of plasma	0.604	< 0.001**
HDL phospholipids (mg/dL) – Apo A1 (ng/ml)	0.524	< 0.001**
HDL phospholipids (mg/dL) – Apo B (ng/ml)	−0.174	0.009*
HD -phospholipids (mg/dL) – Apo B/A1 (ng/ml)	−0.586	< 0.001**

ASCVD, atherosclerotic cardiovascular disease; HDL, high-density lipoprotein; LDL, low-density lipoprotein; *p*-value, level of significant; *r*, correlation coefficient; Apo, apolipoprotein.

* , correlation is significant at the 0.01 level;

** , correlation is significant at the 0.05 level.

Apo B was positively correlated with atherogenic lipids and lipoproteins (triglycerides, TC, LDLC, and non-HDLC). In addition, the Apo B/A1 ratio exhibited strong positive correlations with conventional lipid indices, including CRI-I (*r* = 0.626, *p* < 0.001), CRI-II (*r* = 0.601, *p* < 0.001), AC (*r* = 0.613, *p* < 0.001), and AIP (*r* = 0.604, *p* < 0.001). High-density lipoprotein phospholipid levels correlated positively with Apo A1 (*r* = 0.524, *p* < 0.001) but negatively with Apo B (*r* = –0.174, *p* = 0.009) and the Apo B/A1 ratio (*r* = –0.586, *p* < 0.001).

### Variations in the concentrations of Apo A1, Apo B, and the Apo B/A1 ratio across atherosclerotic cardiovascular disease subclasses

**FIGURE 1:**
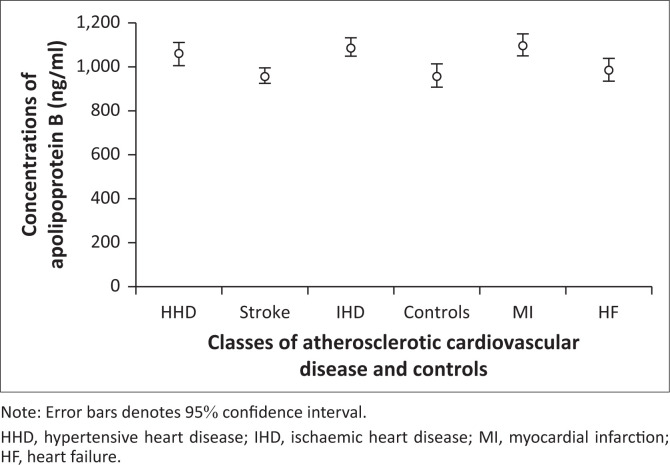
Graded variations in the mean plasma apolipoprotein B concentrations in atherosclerotic cardiovascular disease subclasses and controls at Lagos State University Teaching Hospital, Lagos, Nigeria, between November 2021 and November 2022.

**FIGURE 2:**
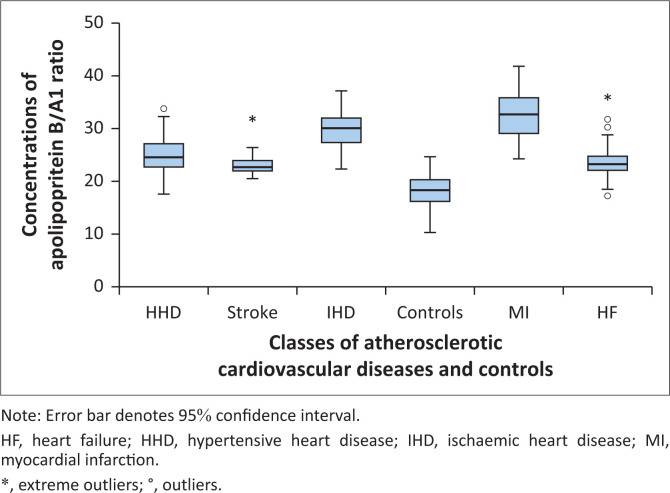
Graded variations in apolipoprotein B/A1 ratio in atherosclerotic cardiovascular disease subclasses and controls at Lagos State University Teaching Hospital, Lagos, Nigeria, between November 2021 and November 2022.

**FIGURE 3:**
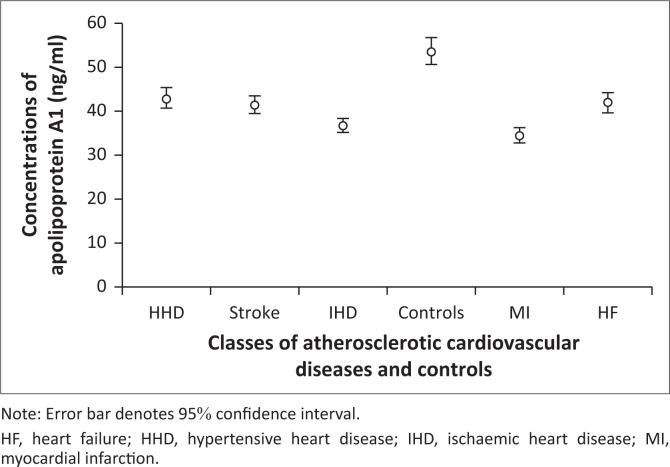
Graded variations in the mean plasma apolipoprotein A1 levels in atherosclerotic cardiovascular disease subclasses and controls at Lagos State University Teaching Hospital, Lagos, Nigeria, between November 2021 and November 2022.

Graded variations were observed in mean plasma Apo B concentrations across ASCVD subclasses (Figure 1), with the highest levels found in MI patients. Similarly, the mean plasma Apo B/A1 ratio showed a graded increase (Figure 2), peaking in IHD patients. In contrast, mean plasma Apo A1 levels exhibited a graded decline (Figure 3), with the lowest levels recorded in MI patients.

### Comparison of the total and high-density lipoprotein phospholipids levels across different classes of atherosclerotic cardiovascular disease

**FIGURE 4:**
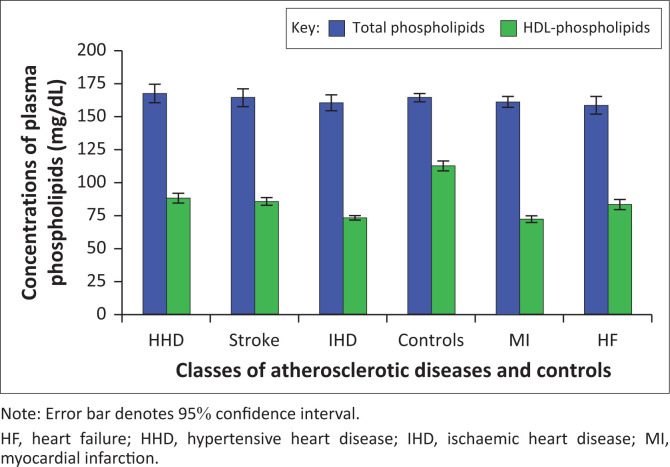
Plasma total and high-density lipoprotein phospholipids levels in atherosclerotic cardiovascular disease subclasses and controls at Lagos State University Teaching Hospital, Lagos, Nigeria, between November 2021 and November 2022.

The mean total plasma phospholipid levels remained within a narrow range across ASCVD patients and controls (Figure 4), with no significant difference between the groups (Table 1). However, the mean plasma HDL phospholipid fraction was significantly higher in controls than in ASCVD patients, with the lowest levels observed in those with MI and IHD.

## Discussion

The present study demonstrated significant disruptions in atherogenic lipids and lipoproteins among ASCVD patients. Notably, there were substantial increases in plasma triglycerides, TC, LDLC, and non-HDLC levels, whereas HDLC (an antiatherogenic lipid) was markedly reduced compared with the control values. Similarly, conventional lipid parameters (CRI-I, CRI-II, AC, and AIP) were markedly elevated in ASCVD patients compared with controls. These findings are consistent with earlier studies,^20,39^ reporting elevated levels of atherogenic lipids and lipid indices in cardiovascular disease patients.

In addition, this study revealed significant variations in lipid profiles and indices across different subclasses of ASCVD. Notably, the greatest elevations in atherogenic lipid and lipid indices were observed in patients with myocardial infarction and IHD. Conversely, these groups also presented the lowest reduction in antiatherogenic HDL cholesterol levels compared with the other ASCVD subclasses. These findings align with previous reports demonstrating severe dyslipidaemia in patients with MI and IHD compared with other ASCVD subtypes.^8,40^ The results of this study further underscore the pronounced severity of dyslipidaemia in these patient groups.

Furthermore, this study revealed a substantial decrease in the mean plasma Apo A1 concentration, and notable increases in Apo B levels and the Apo B/A1 ratio in ASCVD patients, compared with those in the control group. These findings are consistent with prior research across diverse populations worldwide,^13,14,16,17^ which revealed that low Apo A1 levels, elevated Apo B levels, and a higher Apo B/A1 ratio are associated with a heightened risk of major cardiovascular events.

Elevated Apo B levels in ASCVD patients probably indicate an increased burden of atherogenic particles, which is correlated with increased cardiovascular risk.^38^ Concurrently, a higher Apo B/A1 ratio suggests an imbalance favouring atherogenic particles over antiatherogenic particles, highlighting an adverse lipid profile in the ASCVD group.^16^

Notably, the lowest reduction in plasma Apo A1 levels and the greatest increases in both Apo B levels and the Apo B/A1 ratio were observed in patients with MI and IHD. Thus, these findings underscore the critical role of Apo B in the manifestation of MI and IHD, as elevated levels of Apo B-containing lipoproteins primarily drive atherosclerosis.^38^ Moreover, reduced Apo A1 levels in these ASCVD subclasses (MI and IHD) suggest impaired reverse cholesterol transport, which may contribute to the lipid build-up and instability of plaques.^13^ Plaque rupture is a common cause of MI,^41^ making Apo A1 a crucial factor in these patients.

Furthermore, the present study revealed a marked reduction in the mean HDL phospholipid levels in ASCVD patients compared with those in the controls. This finding is in agreement with previous studies that reported a reduction in HDL phospholipid levels in ASCVD patients compared with those in the control group.^23,26^ These findings suggest a potential impairment in HDL functionality, which could contribute to the pathophysiology of ASCVD.

Moreover, the reduction in HDL phospholipid levels was particularly pronounced in patients suffering from MI and IHD. These findings could imply that these ASCVD subclasses are associated with more severe alterations in lipid metabolism and HDL function, possibly exacerbating the risk and severity of ASCVD.

In addition, the present study revealed significant inverse correlations between Apo A1 levels and atherogenic lipids (TC, triglycerides, LDLC, and non-HDLC), and a positive association with HDLC, suggesting a protective effect against atherosclerosis. Conversely, elevated levels of Apo B correlate with increased levels of atherogenic particles, indicating increased cardiovascular risk.

This study also identified the Apo B/A1 ratio as a comprehensive marker for lipid-related ASCVD risk, indicating that it is a superior marker for ASCVD stratification and demonstrating a strong correlation with traditional lipid indices. Higher ratios were associated with elevated levels of atherogenic lipids and decreased levels of HDLC, effectively integrating both proatherogenic and antiatherogenic lipid particles. This ratio provides a more complete view of an individual’s lipid profile and their associated cardiovascular risk.^16,17^

### Strengths, contributions, and implications for future research

The findings of this study carry significant clinical and public health relevance for Nigerian and sub-Saharan African populations. The elevated apolipoprotein B/A1 ratio, coupled with changes in apolipoprotein A1, apolipoprotein B, and HDL phospholipids, indicates a higher risk of premature ASCVD. The apolipoprotein B/A1 ratio emerges as a key marker for ASCVD risk, reinforcing the need for earlier diagnosis and targeted interventions. The findings also underscore the importance of early screening and prevention programmes tailored to these regions.

Future research should focus on tracking changes in the apolipoprotein B/A1 ratio over time and assessing targeted interventions for ASCVD prevention in sub-Saharan Africa. Developing tailored screening programmes and investigating genetic and environmental factors influencing ASCVD risk are crucial. Comparative studies across sub-Saharan Africa can refine risk assessment, while public health initiatives and policy research may help to reduce ASCVD mortality in these regions.

### Limitations

While the findings provide important insights, certain factors warrant consideration. Unmeasured variables such as diet, physical activity and genetic predisposition may have influenced apolipoprotein and HDL phospholipid levels. The mean age difference of about 10 years between cases and controls, despite statistical adjustment, may also introduce residual confounding. In addition, the moderate areas under curve observed in preliminary analyses for Apo A1, Apo B, and HDL phospholipids probably reflect the sample size and exploratory design of the study. Further research with larger, diverse cohorts is needed to validate these findings and to refine clinically relevant cut-off values through advanced modelling approaches.

### Conclusion

This study identified associations between lipid profile disruptions and ASCVD in Nigerian patients, characterised by elevated atherogenic lipids (triglycerides, TC, LDLC, and non-HDLC) and reduced HDLC, particularly in those with MI and IHD. Apolipoprotein analysis showed decreased Apo A1 and increased Apo B, leading to a higher Apo B/A1 ratio and indicating a greater atherogenic burden. Reduced HDL phospholipid levels further suggest impaired HDL functionality. The Apo B/A1 ratio emerged as a key marker for lipid-related ASCVD risk, highlighting its potential for early diagnosis and risk stratification. These findings underscore the need for targeted screening and prevention strategies to improve cardiovascular outcomes in this population.
